# Comparison of menstrual cycle irregularities among young women based on coronavirus disease 2019 infection status: a cross-sectional study

**DOI:** 10.1590/1806-9282.20230801

**Published:** 2024-03-04

**Authors:** Elif Doğan, Betül Uncu, Rukiye Duman

**Affiliations:** 1 Istanbul University-Cerrahpasa, Faculty of Health Science, Department of Midwifery – İstanbul, Turkey.

**Keywords:** COVID-19, SARS-CoV-2, Menstrual cycle, Pandemic, Side effects

## Abstract

**OBJECTIVE:**

The coronavirus disease 2019 pandemic that has emerged recently has significantly affected and continues to affect our lives. The Severe Acute Respiratory Syndrome Coronavirus 2 virus has significant effects on women’s health due to gender-related physiological differences. The aim of this study was to compare the menstrual cycle status of young women according to their status of having had coronavirus disease 2019.

**METHODS:**

This descriptive cross-sectional study was conducted with 220 young women aged between 18 and 25 years who received at least one dose of coronavirus disease 2019 vaccine. The study data were collected as a survey on the online platform.

**RESULTS:**

The descriptive characteristics of young women who had had and had not had coronavirus disease 2019 were distributed homogeneously between the groups (p>0.05). Furthermore, there was no statistical difference in terms of menstrual cycle patterns (p>0.05). The mean scores from the Premenstrual Syndrome Scale and its subscales and the mean scores from the COVID Stress Scale and its subscales were similar in both groups, and no statistically significant difference was identified (p>0.05).

**CONCLUSION:**

Although menstrual cycle irregularities due to coronavirus disease 2019 have been reported, these effects are usually observed during the pandemic. A decrease in stress and anxiety with the end of the pandemic may explain the return of the menstrual cycle to normal.

## INTRODUCTION

The COVID-19 pandemic that has emerged recently has significantly affected and continues to affect our lives^1^. Hence, studies to elucidate the short- and long-term effects of COVID-19 on the human body are increasing^2,3^. During the pandemic, the prevention, diagnosis, and treatment process have been discussed on a global scale, and vaccine development studies have been initiated. Vaccine research has yielded positive results, and vaccines that ensure protection against the SARS-CoV-2 virus and considerably reduce fatal complications have been produced^4^. Nevertheless, just as complications occur in individuals who have had COVID-19, some side effects are also reported after vaccination. These side effects include mild symptoms, such as fever, shivering, headache, fatigue, and arm pain, and life-threatening conditions, such as nausea and stroke^5^. The effects of the SARS-CoV-2 virus on women’s health are an important issue due to gender-related physiological differences^6^. Viruses such as hepatitis B and C and HIV can affect the reproductive system and cause menstrual cycle disorders^7^. It is thought that COVID-19 specifically affects sex hormones and therefore causes changes in the menstrual cycle^8,9^.

Menstrual bleeding, which has a dynamic and cyclic character, is one of the objective markers of women’s health and fertility^10^. An irregular menstrual cycle leads to an increased risk of death in women under the age of 70 years and causes metabolic diseases such as diabetes and dyslipidemia^11,12^.

The International Federation of Gynecology and Obstetrics (FIGO) defines a normal menstrual cycle as the cycle that occurs every 24–38 days, lasts 8 days or less each time, occurs at regular intervals each time, and ends with blood loss that a woman describes as ‘normal’^13^. When COVID-19 vaccines were first administered, there were concerns that menstrual cycle disorders might occur in women due to vaccines^14^. Menstrual cycle irregularities are more common in young women^15^, and even cycle abnormalities due to COVID-19 in the said population are reported in the literature^16^.

Menstrual cycle irregularities due to COVID-19 have first started to draw attention through the media^16^. Subsequent studies and published reports have supported this. Changes in menstrual cycle length and severity have been observed after the COVID-19 vaccine^17^. According to the results of a retrospective study, it is stated that SARS-CoV-2 infection and the COVID-19 vaccine may affect the menstrual cycle^18^. In a report of the United States Vaccine Adverse Event Reporting System involving 3,959 women (2,403 vaccinated and 1,555 unvaccinated), it is indicated that women who have received the COVID-19 vaccine have at least a 1-day extension in their menstrual cycles compared to their cycles before vaccination. No significant change has been recorded in unvaccinated women^19^. According to a systematic review conducted in 2022, decreased bleeding amount and prolonged menstrual cycle length are reported in women who have had COVID-19. Furthermore, according to the study, the severity of COVID-19 does not affect the menstrual cycle^20^. Despite the presence of important studies on the effect of COVID-19 on the female reproductive system and changes in the menstrual cycle, the data available to date are not robust enough to draw definite conclusions on the subject. There was no study comparing the young female population concerning COVID-19 within the scope of the literature accessed. It was found that studies in the literature investigating menstrual cycle irregularities due to COVID-19 were conducted during the period when the virus was active. Accordingly, it is thought that the study conducted during the period when the vaccine became widespread and reduced the effect of the virus will contribute to the literature.

The objective of this study is to compare menstrual cycle irregularities in young women due to the status of having had COVID-19.

## METHODS

### Objective

This research was designed as a descriptive and cross-sectional study to compare the menstrual cycle status of young women between the ages of 18 and 25 years who have received at least one dose of the COVID-19 vaccine according to their status of having had COVID-19.

### Sample

**Figure 1:**
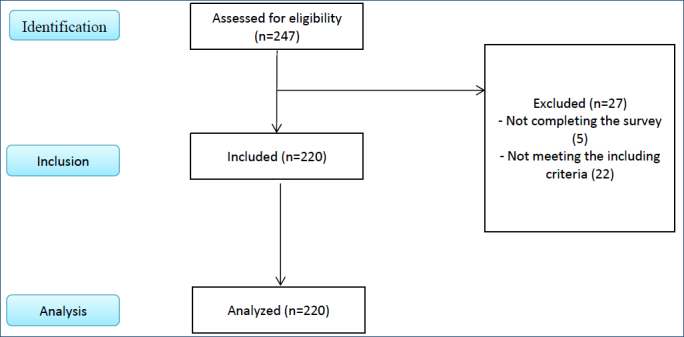
Strengthening the Reporting of Observational Studies in Epidemiology flow diagram.

The study sample consisted of young women between the ages of 18 and 25 years who were students at a university’s faculty of health sciences between January 2022 and March 2023 and met the inclusion criteria. The program G*Power (3.1.9.2) was used to determine the sample size with a power of 90%, a margin of error of 0.05, and an effect level of 0.25. Accordingly, a total of 220 women, including 110 women who had had COVID-19 but were vaccinated and 110 women who had not had COVID-19 but were vaccinated, were planned to be included in the study. The flowchart was prepared in line with the Strengthening the Reporting of Observational Studies in Epidemiology (STROBE) flow diagram^21^ (Figure 1).

Inclusion criteria were as follows: women aged between 18 and 25 years, who received at least one dose of the COVID-19 vaccine.

Exclusion criteria were as follows: women who had a chronic disease, who were diagnosed with obstetric or gynecological disease, and who were pregnant

### Assessment tools

The Menstrual Cycle Information Form, prepared in line with the literature, was used to collect data. The classification of the menstrual cycle pattern used in this form was made according to the FIGO’s classification of normal and abnormal uterine bleeding symptoms^13^.

#### Premenstrual Syndrome Scale

The scale developed by Gençdoğan aims to measure the severity of premenstrual symptoms. The lowest score that can be obtained from the scale is 44, and the highest score is 220. The severity of premenstrual symptoms increases with the increasing score on the scale^22^. Cronbach’s alpha coefficient was 0.75 in the original version of the scale and 0.78 in this study.

#### COVID Stress Scale

This scale was developed by Taylor et al.^23^ to evaluate the stress caused by COVID-19 in a multidimensional manner. The lowest score that can be obtained from the scale is 36, and the highest score is 180. The severity of the stress individuals experience regarding the pandemic increases with the increasing score from the scale^24^. While Cronbach’s alpha coefficient of the scale is 0.97, it was found to be 0.89 in the current study.

### Data collection

The data of this study were collected in the form of a survey on the online platform.

### Statistical analysis

The SPSS (Statistical Package for the Social Sciences) version 25.0 package program was used in the statistical analysis of the data. Continuous variables were presented as arithmetic mean $\bar{\text{X}}$ and standard deviation (SD), and categorical variables were presented as number (n) and percentage (%). The Shapiro-Wilk test was conducted to evaluate the suitability of continuous variables for normal distribution. All continuous variables were nonparametric. The Mann-Whitney U test was applied for nonparametric continuous variables compared between the groups.

### Ethics of the study

The Clinical Research Ethics Committee of the hospital where the study was conducted provided ethical approval for this study (571406/22.12.2022). Informed consent was obtained from all participants who agreed to participate in the study. All stages of the study were conducted in accordance with the Declaration of Helsinki.

## RESULTS

Table 1 contains data on the characteristics and comparison of menstrual cycle patterns of women who have had and have not had COVID-19. Accordingly, 81.8% of women who have had COVID-19 have menstruation every 24–38 days, 91.8% have bleeding for less than 9 days, and 70% have a menstrual cycle pattern with a moderate amount of bleeding. While 80.9% of these young women have a deviation in the menstrual cycle of 10 days or less, 85.5% do not experience breakthrough bleeding at all.

**Table 1:** Comparison of the menstrual cycle characteristics of women who have had and have not had coronavirus disease 2019.

	Women who have had COVID-19 (n=110)	Women who have not had COVID-19 (n=110)	z	p
	% (n)	% (n)		
Menstrual cycle frequency			0.939	0.625
<24 days	12.7 (14)	17.3 (19)		
≥24 to ≤38 days	81.8 (90)	78.2 (86)		
>38 days	5.5 (6)	4.5 (5)		
Menstrual cycle regularity			1.196	0.274
≤7–9 days	80.9 (89)	86.4 (95)		
≥10 days	19.1 (21)	13.6 (15)		
Menstrual cycle duration			2.044	0.153
≤8 days	91.8 (101)	96.4 (106)		
≥9 days	8.2 (9)	3.6 (4)		
Menstrual bleeding volume			0.263	0.877
Light (<5 mL)	9.1 (10)	10.9 (12)		
Normal (≥5 to ≤80 mL)	70 (77)	67.3 (74)		
Heavy (>80 mL)	20.9 (23)	21.8 (24)		
Intermenstrual bleeding			1.595	0.450
None	85.5 (94)	79.1 (87)		
Random	13.6 (15)	20.0 (22)		
Cyclic	0.9 (1)	0.9 (1)		

Z: Mann-Whitney U test.

Of the women who have not had COVID-19, 78.2% have menstruation every 24–38 days, 94.4% have bleeding for less than 9 days, and 67.3% have a menstrual cycle pattern with a moderate amount of bleeding. While 86.4% of these young women experience a deviation in the menstrual cycle of 10 days or less, 79.1% do not experience breakthrough bleeding at all. Upon comparing both groups according to menstrual cycle characteristics, no statistically significant difference was found in all subcharacteristics (p>0.05) (Table 1).

Table 2 presents data comparing the Premenstrual Syndrome Scale (PSS) and COVID Stress Scale (CSS) mean scores of women who have had and have not had COVID-19. Accordingly, young women who had had COVID-19 received the following mean scores from the subscales of the PSS: 24.3±0.7 from the “Depressive Mood” subscale, 19.4±0.7 from the “Anxiety” subscale, 22.4±0.6 from the “Fatigue” subscale, 17.1±0.6 from the “Irritability” subscale, 21.5±0.8 from the “Depressive Thoughts” subscale, 9.5±0.3 from the “Pain” subscale, 10.7±0.3 from the “Appetite Changes” subscale, 8.9±0.3 from the “Sleeping Changes” subscale, and 9.5±0.4 from the “Bloating” subscale. The mean total score of 143.4±3.9 was obtained from the scale.

**Table 2:** Comparison of the Premenstrual Syndrome Scale and COVID Stress Scale scores of women who have had and have not had coronavirus disease 2019.

	Women who have had COVID-19 (n=110)	Women who have not had COVID-19 (n=110)	z	p
	Mean±SD	Mean±SD		
PSS subthemes				
Depressive mood	24.3±0.7	23.9±0.7	-0.799	0.424
Anxiety	19.4±0.7	18.9±0.7	-0.450	0.653
Fatigue	22.4±0.6	21.6±0.5	-1.202	0.229
Irritability	17.1±0.6	16.7±0.5	-0.580	0.562
Depressive thoughts	21.5±0.8	20.2±0.7	-1.522	0.128
Pain	9.5±0.3	9.5±0.3	-0.022	0.982
Appetite changes	10.7±0.3	10.3±0.3	-0.897	0.370
Sleeping changes	8.9±0.3	9.0±0.3	-0.171	0.864
Bloating	9.5±0.4	8.7±0.3	-1.415	0.157
Total PSS score	143.4±3.9	138.9±3.6	-1.280	0.201
CSS subthemes				
Threat/danger	19.1±0.6	18.7±0.6	-0.377	0.706
Socioeconomic results	10.4±0.5	9.8±0.4	-0.739	0.460
Xenophobia	13.8±0.6	14.5±0.6	-0.762	0.444
Contamination	16.6±0.6	16.7±0.6	-0.073	0.942
Traumatic stress	10.9±0.5	11.2±0.5	-0.765	0.444
Compulsive control	15.8±0.6	14.3±0.6	-1.660	0.097
Total CSS score	86.7±2.6	85.3±2.5	-0.424	0.672

Z: Mann-Whitney U test.

Young women who had not had COVID-19 obtained the following mean scores from the PSS subscales: 23.9±0.7 from the “Depressive Mood” subscale, 18.9±0.7 from the “Anxiety” subscale, 21.6±0.5 from the “Fatigue” subscale, 16.7±0.5 from the “Irritability” subscale, 20.2±0.7 from the “Depressive Thoughts” subscale, 9.5±0.3 from the “Pain” subscale, 10.3±0.3 from the “Appetite Changes” subscale, 9.0±0.3 from the “Sleeping Changes” subscale, and 8.7±0.3 from the “Bloating” subscale. The mean total score of 138.9±3.6 was obtained from the scale. No statistically significant difference was revealed between the groups in terms of the mean PSS score and PSS subscale mean scores (p>0.05) (Table 2).

The mean scores obtained by women who have had COVID-19 from the CSS subscales are as follows: 19.1±0.6 from the “Threat/danger” subscale, 10.4±0.5 from the “Socioeconomic results” subscale, 13.8±0.6 from the “Xenophobia” subscale, 16.6±0.6 from the “Contamination” subscale, 10.9±0.5 from the “Traumatic stress” subscale, and 15.8±0.6 from the “Compulsive control” subscale. The mean total score obtained from the scale is 86.7±2.6.

Young women who have not had COVID-19 received the following mean scores from the CSS subscales: 18.7±0.6 from the “Threat/danger” subscale, 9.8±0.4 from the “Socioeconomic results” subscale, 14.5±0.6 from the “Xenophobia” subscale, 16.7±0.6 from the “Contamination” subscale, 11.2±0.5 from the “Traumatic stress” subscale, and 14.3±0.6 from the “Compulsive control” subscale. The mean total score of 85.3±2.5 was obtained from the scale. No statistically significant difference was found between the groups in terms of the mean CSS score and CSS subscale mean scores (p>0.05) (Table 2).

## DISCUSSION

This study determined no difference in terms of menstrual cycle changes between women who had had and had not had COVID-19. A study comparing women who had had COVID-19 in the last 6 months and those who had never had it reported menstrual cycle disorders in women who had had COVID-19^25^. A study examining 177 women who had had COVID-19 and had menstrual cycle records similarly reported menstrual cycle changes in these women^9^. According to a systematic review, although COVID-19 was initially regarded to prolong the menstrual cycle length and cause changes in the amount of menstrual bleeding, the disease severity had no effect on menstrual cycle changes^20^. It is known that menstrual cycle disorders have increased in women due to increased stress and anxiety during the pandemic. A systematic review reported an increase in menstrual symptoms and a prolongation of the menstrual cycle length in women who had had COVID-19^26^. Ozimek et al.^27^ reported that increased menstrual irregularities with the pandemic were associated with women’s increased stress levels. A study involving 1,031 women showed that the pandemic significantly affected women’s reproductive health. According to the study, 46% of women experienced menstrual cycle changes and problems and these changes included problems such as menorrhagia, dysmenorrhea, and changes in the length of menstrual cycle^28^. Another study revealed changes in the menstrual cycle during the pandemic and an increase in dysmenorrhea^29^. Likewise, the results of a study conducted with 1,335 women reported that 56% of women had menstrual cycle changes and dysmenorrhea during the pandemic^30^. A study conducted in 2021 suggested that even if you had not had COVID-19, the pandemic itself caused an increase in anxiety and stress levels, which impacted menstrual cycle characteristics^31^. As mentioned above, having had COVID-19 may cause menstrual cycle changes and irregularities in women. However, as this pandemic has occurred recently, it is important to research its long-term effects with current studies. Li et al.^9^ examined menstrual cycle changes in women who had had COVID-19 infection in the last 6 months. According to the study results, menstrual cycle changes lasted up to 3 months at most. Another study detected menstrual cycle changes in women with higher perceived stress during the pandemic^32^. According to our study findings, no significant difference was revealed between the groups in terms of the CSS total score and subscale scores. The similar menstrual cycle patterns of the participants can be explained by similar stress levels. According to the same study, regardless of stress levels, the irregularity of the menstrual cycle in women over the age of 40 years, depending on whether they had had COVID-19, was more common than in women under the age of 40 years^32^. Participants of the current study were between the ages of 18–25 years. The fact that the study was conducted with a young population may explain why the menstrual cycle was not affected by the status of having had COVID-19. In a recent study carried out in 2023, menstrual cycle changes decrease as the time since COVID-19 infection increases^33^. Another recent study reported that menstrual cycle changes were temporary^34^. Considering that the data of this study were collected in 2023, it is thought that the majority of the participants who had had COVID-19 had infection during the pandemic. Therefore, menstrual cycle changes can be expected to be similar to the group who had not had the infection.

According to the findings we obtained, the status of having had COVID-19 infection did not cause menstrual cycle changes. It is considered to be associated with the end of the pandemic at the time when the study was conducted and the return to normal life. It is thought that the time period of the studies in the literature coincided with the most active period of the pandemic, which ensured that the menstrual cycle changed with increasing anxiety levels.

## CONCLUSION

Although there is information in the literature that menstrual cycle disorders develop due to COVID-19, this can be explained by the fact that the increased anxiety and stress during the pandemic affect the menstrual cycle pattern. As there is no study in the literature investigating menstrual cycle characteristics due to COVID-19 in young women in the postpandemic period, this study is unique and more studies are needed.

### Limitation

The study data were obtained during the period when the SARS-CoV-2 virus was taken under control and vaccination became widespread. Data concerning the menstrual period questioned the present, not the past, which is thought to have affected the results. The study was conducted only on young women aged between 18 and 25 years and can be repeated in different age groups. Another limitation of the study is that women in the sample group have received the COVID-19 vaccine. Results may differ in women who have not been vaccinated. The study was performed on individuals who had received at least one dose of the vaccine. The effect of the number of vaccine doses on the menstrual cycle has not been mentioned.

## ABBREVIATIONS

COVID-19: coronavirus disease 2019
SARS-CoV-2: severe acute respiratory syndrome coronavirus 2
FIGO: The International Federation of Gynecology and Obstetrics
CSS: COVID Stress Scale
PSS: Premenstrual Syndrome Scale
STROBE: Strengthening the Reporting of Observational Studies in Epidemiology
