# Erratum

**DOI:** 10.1590/1806-9282.20230801ERRATUM

**Published:** 2025-03-31

In the manuscript "Comparison of menstrual cycle irregularities among young women based on coronavirus disease 2019 infection status: a cross-sectional study", https://doi.org/10.1590/1806-9282.20230801, published in the Rev Assoc Med Bras. 2024;70(2):e20230801, on page 2:

**Where it reads:**

The study sample consisted of young women between the ages of 18 and 25 years who were students at a university's faculty of health sciences between January 2022 and March 2023 and met the inclusion criteria.

**It should read:**

The study sample consisted of young women between the ages of 18 and 25 years who were students at a university's faculty of health sciences between January 2023 and March 2023 and met the inclusion criteria.

